# Estimating the Prevalence and Awareness Rates of Hypertension in Africa: A Systematic Analysis

**DOI:** 10.1371/journal.pone.0104300

**Published:** 2014-08-04

**Authors:** Davies Adeloye, Catriona Basquill

**Affiliations:** Centre for Population Health Sciences, University of Edinburgh Medical School, Edinburgh, Midlothian, United Kingdom; University Heart Center, Germany

## Abstract

**Background:**

The burden of hypertension is high in Africa, and due to rapid population growth and ageing, the exact burden on the continent is still far from being known. We aimed to estimate the prevalence and awareness rates of hypertension in Africa based on the cut off “≥140/90 mm Hg”.

**Methods:**

We conducted a systematic search of Medline, EMBASE and Global Health. Search date was set from January 1980 to December 2013. We included population-based studies on hypertension, conducted among people aged ≥15 years and providing numerical estimates on the prevalence of hypertension in Africa. Overall pooled prevalence of hypertension in mixed, rural and urban settings in Africa were estimated from reported crude prevalence rates. A meta-regression epidemiological modelling, using United Nations population demographics for the years 1990, 2000, 2010 and 2030, was applied to determine the prevalence rates and number of cases of hypertension in Africa separately for these four years.

**Results:**

Our search returned 7680 publications, 92 of which met the selection criteria. The overall pooled prevalence of hypertension in Africa was 19.7% in 1990, 27.4% in 2000 and 30.8% in 2010, each with a pooled awareness rate (expressed as percentage of hypertensive cases) of 16.9%, 29.2% and 33.7%, respectively. From the modelling, over 54.6 million cases of hypertension were estimated in 1990, 92.3 million cases in 2000, 130.2 million cases in 2010, and a projected increase to 216.8 million cases of hypertension by 2030; each with an age-adjusted prevalence of 19.1% (13.9, 25.5), 24.3% (23.3, 31.6), 25.9% (23.5, 34.0), and 25.3% (24.3, 39.7), respectively.

**Conclusion:**

Our findings suggest the prevalence of hypertension is increasing in Africa, and many hypertensive individuals are not aware of their condition. We hope this research will prompt appropriate policy response towards improving the awareness, control and overall management of hypertension in Africa.

## Introduction

Hypertension and other cardiovascular diseases rank among the leading causes of disabilities and deaths from non-communicable diseases (NCDs) in Africa [1], [2], with rising prevalence and death rates now observed more in young and active adults [3]. Recently, the African Union (AU) reported that hypertension is one of the greatest health challenges after HIV/AIDS in the continent [4]. This is in fact a priority globally as conclusions from the 2011 United Nations high level meeting on NCDs focus on a reduction of hypertension and other NCDs, especially in Africa, where the burden is rising at a faster rate compared to other parts of the world [5]. Worldwide, cardiovascular diseases account for about 17 million deaths, with complications from poorly controlled hypertension resulting in over 7.5 million deaths and 57 million disability adjusted life years (DALYS) [6].

The relatively higher prevalence of hypertension in Africa has been linked to population growth and ageing, rising urbanization, mass migration from rural to urban areas, and an increased uptake of western lifestyles including tobacco and alcohol consumption [3], [7]. Public health response from the governments of many African nations still remains low, as research findings show that a high number of hypertensive individuals are currently unaware of their condition [8]. Many African countries are yet to implement high blood pressure awareness and control programmes on a population-wide scale, even to people with very high risk of cardiovascular complications [9]. Even with a widely popularised availability of hypertension treatments in some African countries [10], reports show that many rural dwellers are still faced with lack of antihypertensive medications and poor management of cases of hypertension [11]. The WHO has recommended a need for strong public health policies, multisectoral approach, and available and affordable treatment options toward reducing this growing burden of hypertension in Africa [2].

Despite reports of a higher prevalence of hypertension in Africa compared to other world regions [2], public health experts believe the real burden is still far from being known [12]. Many studies in the 1980s and early 1990s were based on the old definition of hypertension (≥160/95 mm Hg) [8], [12]. These surveys may possibly underestimate the prevalence of hypertension in Africa in comparison to newer surveys based on ≥140/90 mm Hg. Moreover, even within studies based on similar case definitions, the variations in reported estimates still suggest the need for more systematic and accurate estimates from larger number of studies towards appropriately informing health service planning and a better response to hypertension in Africa. For example, the World Health Organization (WHO) reported that the prevalence of hypertension in the African region was highest globally in 2008, with an estimated prevalence of 46% [13]. This, though vital for instituting relevant public health response in the continent, elucidates a conflicting state of data in comparison to other hypertension prevalence estimates in Africa which are relatively lower [14], [15]. Thus, with an increased research output on hypertension in Africa in the last two decades, a systematic review of population-based studies was conducted towards providing an improved continent-wide estimate of the prevalence and awareness rate of hypertension in Africa, which hopefully may encourage healthy public health policy for a value-added management of hypertension in the region.

## Methods

### Search strategy

After identifying Medical Subject Headings (MESH) and keywords, a final search strategy was developed. Searches were conducted on three main databases: Medline, EMBASE and Global Health. The search date was set from January 1980 to December 2013. African countries were as listed on the World Bank list of economies (July 2012) [16]. See **Table 1** for details of the search terms.

**Table 1:** Search terms.

#	Search terms
**1**	africa/ or africa, northern/ or algeria/ or egypt/ or libya/ or morocco/ or africa, central/ or cameroon/ or central african republic/ or chad/ or congo/ or “democratic republic of the congo”/ or equatorial guinea/ or gabon/ or africa, eastern/ or burundi/ or djibouti/ or eritrea/ or ethiopia/ or kenya/ or rwanda/ or somalia/ or sudan/ or tanzania/ or uganda/ or africa, southern/ or angola/ or botswana/ or lesotho/ or malawi/ or mozambique/ or namibia/ or south africa/ or swaziland/ or zambia/ or zimbabwe/ or africa, western/ or benin/ or burkina faso/ or cape verde/ or cote d'ivoire/ or gambia/ or ghana/ or guinea/ or guinea-bissau/ or liberia/ or mali/ or mauritania/ or niger/ or nigeria/ or senegal/ or sierra leone/ or togo/
**2**	exp vital statistics/ or exp incidence/
**3**	(incidence* or prevalence* or morbidity or mortality).tw.
**4**	(disease adj3 burden).tw.
**5**	exp “cost of illness”/
**6**	exp quality-adjusted life years/
**7**	QALY.tw.
**8**	Disability adjusted life years.mp.
**9**	(initial adj2 burden).tw.
**10**	exp risk factors/
**11**	2 or 3 or 4 or 5 or 6 or 7 or 8 or 9 or 10
**12**	cardiovascular diseases/ or heart diseases/ or exp hypertension/ or peripheral vascular diseases/
**13**	Hypertensive heart disease.mp. [mp = title, abstract, subject headings, heading word, drug trade name, original title, device manufacturer, drug manufacturer, device trade name, keyword]
**14**	12 or 13
**15**	1 and 11 and 14
**16**	limit 15 to (humans and yr = “1980 -Current”)

### Selection criteria and case definitions

Cross-sectional population- and/or community-based studies on hypertension were included, published on or after 1980, conducted among people aged ≥15 years, and providing numerical estimates on the prevalence of hypertension in Africa. Studies conducted before 1980, hospital-based, without numerical estimates, on non-human subjects, and that were mainly reviews were all excluded. There were no language restrictions.

Case definitions of hypertension across retained studies comply with the following:

- systolic blood pressure (≥140 mm Hg) and/or diastolic blood pressure (≥90 mm Hg) and/or self-reported use of antihypertensive medications;
- the sixth and seventh report of the Joint National Committee on prevention, detection, evaluation, and treatment of high blood pressure (JNC 6 & 7) [17], [18]; and
- the 1999 and 2003 World Health Organization/International Society of Hypertension (WHO/ISH) definitions and classification of blood pressure levels [19]

See **Table 2** for details. For the definition of the awareness rate of hypertension in this study, we included studies estimating the prevalence of hypertension based on the definitions above, with awareness rate also being estimated among all identified cases of hypertension and defined as self-report by respondents of any prior diagnosis of hypertension by a doctor or certified health care professional, and excluding women diagnosed during pregnancy, as described by the WHO [19].

**Table 2:** Classification of blood pressure for adults.

Category	JNC				WHO/ISH			
	6		7		1999		2003	
	SBP	DBP	SBP	DBP	SBP	DBP	SBP	DBP
Optimal	<120	and <80	-	-	<120	<80	-	-
Normal	120–129	and 80–84	<120	and <80	<130	<85	-	-
Borderline (JNC)/or High Normal (WHO/ISH)	130–139	or 85–89	120–139 (Pre-hypertension)	or 80–89 (Pre-hypertension)	130–139	85–89	-	-
Hypertension (JNC)/Grade 1 (WHO/ISH)	≥140	or ≥90	≥140	or ≥90	140–159	90–99	140–159	90–99
Stage 1 (JNC)/or Subgroup Borderline (WHO/ISH)	140–159	or 90–99	140–159	or 90–99	140–149	90–94	-	-
Stage 2 (JNC)/or Grade 2 (WHO/ISH)	160–179	or 100–109	≥160 (Stage 2)	or ≥100 (Stage 2)	160–179	100–109	160–179	100–109
Stage 3 (JNC)/or Grade 3 (WHO/ISH)	≥180	or ≥110	-	-	≥180	≥110	≥180	≥110

JNC: Joint National Committee on prevention, detection, evaluation, and treatment of high blood pressure, WHO/ISH: World Health Organization/International Society of Hypertension, SBP: systolic blood pressure, DBP: diastolic blood pressure.

### Quality criteria

Studies retained were assessed for the following quality:

- *Study design*: Under this, flaws in the design and execution of study were examined. Basically, this assesses methods of estimation of sample size and sampling methods across studies, and the methods of dealing with design specific issues such as: training of study investigators, adherence to standardized protocol for blood pressure measurement, pre-testing and reviewing questionnaires before data entry, and addressing recall and interviewer's bias appropriately;
- *Study analysis*: This assessed the appropriateness of statistical and analytical methods employed across studies in the estimation of hypertension prevalence;
- *Generalizability to the African population*: This broadly assessed if the sample size was representative of a larger population that can be generalized to the total African population

An adaptation of the Grading of Recommendations Assessment, Development and Evaluation (GRADE) guidelines was applied in the final quality grading of studies [20]. See **Box S1**, **Table S1** and **Table S2** in **File S1** for details.

### Data extraction and analysis

All extracted data were stored in Microsoft Excel file format. A parallel search and double extraction were conducted by the authors. Data were abstracted systematically on sample size, mean age or age range, number of hypertension cases, and the respective age- and sex-specific prevalence rates. These were sorted into mixed, urban and rural settings (based on studies that reported them). For studies conducted on the same study site, population or cohort, the first chronologically published study was retained, and all additional data from other studies were included in the selected paper.

From all studies, reported crude prevalence rates of hypertension (all expressed as percentages) were pooled, and reported separately for north Africa and sub-Saharan Africa (central, east, south and west), and with rates for urban and rural settings estimated. These were further categorized into 1990, 2000 and 2010, as obtained from studies conducted before 1995, from 1995 to 2004, from 2005 to 2013, respectively. As noted above, awareness rate of hypertension was estimated as number of people who reported being hypertensive, expressed as a percentage of total number of people in the study population having hypertension. Pooled awareness rates of hypertension for 1990, 2000, and 2010, and rates across mixed, urban and rural settings were estimated, respectively.

### Description of the modelling

For the modelling and estimation of the total cases of hypertension in Africa, a meta-regression epidemiological model was developed, and applied on crude prevalence rates, sample sizes and respective mean age from all data points. In this model, all data points (mainly the crude prevalence rates and mean age) were plotted on a graph on Microsoft Excel, with all extracted crude prevalence rates plotted on the *y*-axis, i.e. dependent variable, and mean ages corresponding to each prevalence rate are plotted on the *x*-axis, i.e. independent variable. To account for variation in sample sizes from each data point, bubbles were generated on the graph, with the size of each bubble corresponding to the respective sample size reported. Although, it is well known that the prevalence of hypertension in the population significantly increases with age [21], the relationship between age and the disease may not be necessarily linear. Therefore, we experimented with the models based on linear, logarithmic, exponential, Poisson, polynomial, power function and moving average statistical analyses, respectively, and chose the one which was the most predictive, i.e. in which the proportion of variance (R^2^) of the disease prevalence explained by age was the greatest. On the model therefore, the fitted curve explaining the largest proportion of variance (best fit) was applied. The equation generated from the fitted curve was then used to determine the prevalence of hypertension in Africa at midpoints of the United Nations (UN) population 5-year age-group population estimates for Africa, for the years 1990, 2000, 2010, respectively [22], while an overall model was developed from all data points (1980–2013) and used to predict the number of hypertension cases and prevalence rates for 2030. All statistical analyses were conducted on Microsoft Excel and Stata 13.1 (Copyright 1985–2013 Stata Corp LP).

## Results

### Systematic review

**Figure 1:**
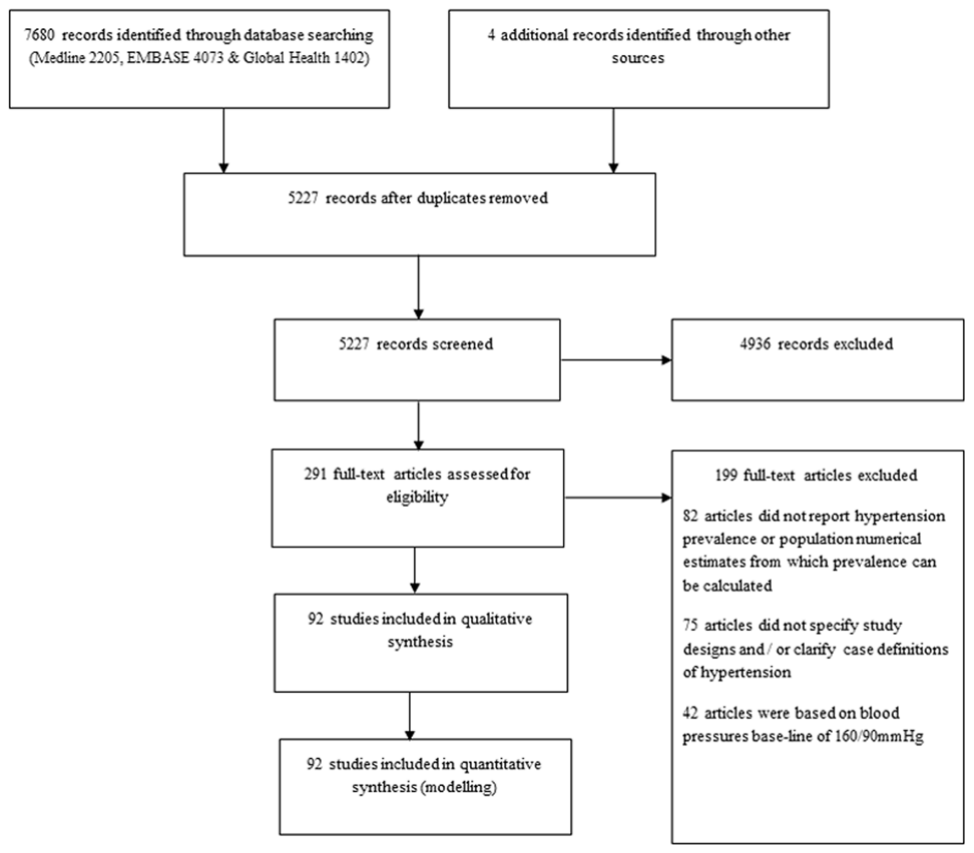
Flow diagram of search results.

The search returned 7680 publications: Medline (2205), EMBASE (4073) and Global Health (1402). An additional 3 studies were included from other sources (Google Scholar and reference list of relevant reviews). After excluding duplicates, 5227 studies remained. On screening titles for relevance (hypertension studies conducted primarily in an African population setting), 4936 articles were excluded, giving a total of 291 full texts that were assessed. 82 articles did not report hypertension prevalence or population denominators from which prevalence rates can be calculated, 75 articles did not specify study designs and/or clarify case definitions of hypertension, and 42 articles were based on blood pressures base-line of 160/90 mm Hg. A total of 92 studies were finally retained for qualitative synthesis and quantitative analysis (**Figure 1**).

### Study characteristics

There were 92 studies conducted across 101 study sites in 31 African countries. Central Africa had 10 study sites, Eastern Africa 21, Northern Africa 12, Southern Africa 15, and Western Africa 43. Nigeria had the highest number of publications with 26 study sites; Ghana and South Africa follow with 7 study sites each, while Cameroon, Tanzania and Tunisia had 6 study sites each (see **Tables 3** **and** **4**).

**Table 3:** Study distribution.

Country	Study sites
*Central*	
Cameroon	6
Chad	1
DR Congo	2
Rwanda	1
*East*	
Eritrea	1
Ethiopia	5
Kenya	3
Seychelles	1
Sudan	1
Tanzania	6
Uganda	4
*North*	
Algeria	3
Egypt	2
Morocco	1
Tunisia	6
*South*	
Angola	2
Madagascar	1
Malawi	2
Mozambique	1
Namibia	1
South Africa	7
Zambia	1
*West*	
Benin	1
Burkina Faso	1
Gambia	1
Ghana	7
Guinea	2
Liberia	1
Nigeria	26
Senegal	2
Togo	2
Duration of study	
<1 year	74
1–3 years	22
>3 years	5
Sample size	
<1000	54
1001–3000	37
>3000	10
Study setting	
Rural	47
Urban	50
Mixed (sites overlap with urban and rural)	33

**Table 4:** Summary of data from all studies.

Country, Setting	Study period	Diagnostic criteria	Mean age (years)	Prevalence % (all)	Prevalence % (men)	Prevalence % (women)
**CENTRAL**						
Cameroon, Mixed [44]	1995	≥140/90 mmHg	49.5	16.9	17.7	16.3
Cameroon, Mixed [45]	1991	≥140/90 mmHg	41.75	7.07	8.92	5.69
Cameroon, Mixed [46]	1994	≥140/90 mmHg	54.5	18.8	20.2	17.8
Cameroon, Mixed [46]	2003	WHO/ISH 1999	54.5	38.34	40.9	36.5
Cameroon, Urban) [47]	2003	WHO/ISH 1999	31.35	24.6	25.6	23.1
Cameroon, Urban [47]	2004	WHO/ISH 1999	31.35	20.8	-	-
Chad, Rural [48]	2004	WHO/ISH 2003	35	16.4	12.2	21.8
DR Congo, Mixed [49]	2009–10	WHO/ISH 2003	54.5	40.2	-	-
DR Congo, Urban [50]	1983–84	≥140/90 mmHg	42.5	16.7	22.1	12.4
Rwanda, Rural [51]	2007	JNC 7	42.2	16.0	16.0	16.0
**EAST**						
Eritea, Mixed [52]	2004	≥140/90 mmHg	39.5	16.0	16.88	15.28
Ethiopia, Mixed [28]	2008	JNC 7, WHO/ISH 2003	36.08	9.9	-	-
Ethiopia, Urban [53]	2012	JNC 7	51.4	28.3	26	30.3
Ethiopia, Urban [54]	2009	≥140/90 mmHg	50.5	19.1	22	14.9
Ethiopia, Urban [55]	2006	≥140/90 mmHg	49.5	30.0	31.5	28.9
Ethiopia, Urban [56]	2009–2010	JNC 7	42.9	17.7	20.0	14.3
Kenya, Rural [57]	2009–11	WHO/ISH 2003	40.9	20.2	-	-
Kenya, Mixed [58]	2007–08	≥140/90 mmHg	69.5	50.1	-	-
Kenya, Urban [59]	2009–09	≥140/90 mmHg	48.5	12.3	12.7	12
Seychelles, Mixed [60]	2004	≥140/90 mmHg	44.5	31.6	38.4	24.8
Sudan, Urban [27]	1988–89	≥140/90 mmHg	35	7.5	-	-
Tanzania, Urban [61]	1998–99	WHO/ISH 1999	54.5	28.9	27.1	30.2
Tanzania, Rural [51]	2007	JNC 7	42.8	27	28	24
Tanzania, Rural [24]	2009–2010	WHO/ISH 2003	76	69.9	62.2	75.8
Tanzania, Rural [62]	1996	WHO/ISH 1999	39.95	29.2	30	28.6
Tanzania, Rural	1996	≥140/90 mmHg	54.5	31.9	32.2	31.5
Tanzania, Urban [57]	2009–11	WHO/ISH 2003	36.8	19	-	-
Uganda, Rural [63]	2008–09	≥140/90 mmHg	32.75	22.3	22.5	22.6
Uganda, Rural [64]	2011	≥140/90 mmHg	42.5	20.5	20.7	20.4
Uganda, Mixed [65]	2012	≥140/90 mmHg	35.15	21.8	22.3	21.7
Uganda, Rural [66]	2006	≥140/90 mmHg	42	30.4	25.4	34
**NORTH**						
Algeria, Rural) [67]	2010	WHO/ISH 2003	58.5	50.2	51.3	49.7
Algeria, Urban [68]	2004–05	≥140/90 mmHg	54.5	32.7	24.5	40.6
Algeria, Peri-urban [69]	2006–07	≥140/90 mmHg	55	44	41.2	46.7
Egypt, Mixed [70]	1991–93	≥140/90 mmHg	45.6	26.3	25.7	26.9
Egypt, Rural [71]	1999–00	≥140/90 mmHg	42.5	27.9	-	-
Morocco, Mixed [72]	2000	≥140/90 mmHg	51	39.6	37.2	41.3
Tunisia, Mixed [73]	2004–05	JNC 7	44.6	31.07	25.0	36.1
Tunisia, Mixed [74]	2004–05	≥140/90 mmHg	49.6	30.6	27.3	33.1
Tunisia, Mixed [75]	2002–03	≥140/90 mmHg	54.5	44.3	38.7	48.2
Tunisia, Urban [76]	1995	≥140/90 mmHg	54.5	28.9	30	28.4
Tunisia, Rural [77]	2008–09	WHO/ISH 2003	72.3	52	45	55.5-
Tunisia, Mixed [25]	2002–03	≥140/90 mmHg	69	69.3	-	-
**SOUTH**						
Angola, Urban [78]	2009–10	JNC 7	44.5	45.2	46.3	44.2
Angola, Mixed [79]	2011	≥140/90 mmHg	41.5	23	26.4	19.8
Madagascar, Urban [80]	1996–97	≥140/90 mmHg	32.75	23.3	24.9	21.7
Malawi, Rural [51]	2007	JNC 7	38.4	23	24.5	22
Malawi, Mixed [81]	2009	≥140/90 mmHg	45.5	33.2	36.9	29.9
Mozambique, Mixed [82]	2005	WHO/ISH 1999	54.5	33.1	35.7	31.2
Namibia, Urban [57]	2009–11	WHO/ISH 2003	36.9	32	-	-
South Africa, Rural [83]	2004–05	≥140/90 mmHg	59.5	28.0	24.5	29.2
South Africa, Rural [84]	2010	≥140/90 mmHg	54.5	26.2	20.8	28.5
South Africa, Mixed [23]	2008	≥140/90 mmHg	65	77.3	74.4	79.6
South Africa, Mixed [85]	1982	≥140/90 mmHg	41	41.6	45.6	37.75
South Africa, Mixed [86]	1990	≥140/90 mmHg	40.5	21.5	19.2	23.4
South Africa, Peri-urban [87]	1996	≥140/90 mmHg	42	27.1	31.9	23.4
South Africa, Rural [88]	2002	JNC 7	59.5	32.6	-	-
Zambia, Urban [89]	2009–10	WHO/ISH 2003	57	34.8	38	33.3
**WEST**						
Benin, Mixed [90]	2008	≥140/90 mmHg	42.7	27.9	-	-
Burkina Faso, Urban [91]	2004	≥140/90 mmHg	54.5	40.2	-	-
Gambia, Mixed [92]	1998–99	≥140/90 mmHg	43.7	18.4	-	-
Ghana, Rural [93]	2004–05	≥140/90 mmHg	42.4	25.4	24.1	25.9
Ghana, Mixed [94]	2004	≥140/90 mmHg	35.9	29.4	31.04	28.07
Ghana, Rural [95]	2003	≥140/90 mmHg	53	32.8	-	-
Ghana, Mixed [96]	2001	≥140/90 mmHg	54.7	28.7	29.9	28
Ghana, Rural [97]	2006–07	≥140/90 mmHg	53.5	35	37.2	34.1
Ghana, Rural [98]	2002–10	JNC 7. WHO/ISH 2003	66	24.1	25.7	22.5
Ghana, Rural [99]	2012	≥140/90 mmHg	53.84	44.7	-	-
Guinea, Mixed [100]	2003	≥140/90 mmHg	62	31.4	-	-
Guinea, Rural [101]	2001	≥140/90 mmHg	45.5	45.2	-	-
Liberia, Rural [102]	1991–92	≥140/90 mmHg	54.5	12.5	-	-
Nigeria, Mixed [103]	2010–11	JNC 6	71.1	34.7	-	-
Nigeria, Semi-urban [104]	2007–08	JNC 7	44.2	36.57	36.79	36.39
Nigeria, Semi-urban [105]	2011–12	JNC 7, WHO/ISH 2003	41.5	25.2	24.7	24.7
Nigeria, Rural [106]	2010–11	≥140/90 mmHg	57.3	44.5	49.3	42.3
Nigeria, Rural [107]	2012–13	JNC 7	41.3	20.2	20.5	20.1
Nigeria, Urban [108]	2006–10	JNC 7	41.9	33	38.3	27.8
Nigeria, Mixed [109]	2008	JNC 7	48.7	50.5	52	49.3
Nigeria, Rural [110]	2011	JNC 7	49.7	13.2	15	11.9
Nigeria, Urban [111]	1987–88	≥140/90 mmHg	36.35	31.1	34	17
Nigeria, Mixed [44]	1995	≥140/90 mmHg	49.5	14.5	14.7	14.3
Nigeria, Rural [112]	2005–06	WHO/ISH 2003	59.8	46.4	50.2	44.8
Nigeria, Semi-urban [113]	2012	≥140/90 mmHg	31.7	47	30.1	16.8
Nigeria, Mixed [114]	2009	≥140/90 mmHg	34.9	21.1	-	-
Nigeria, Semi-urban [115]	2002–03	JNC 6, WHO/ISH 1999	55	21	23.3	16.4
Nigeria, Rural [57]	2009–11	WHO/ISH 2003	45.3	21	-	-
Nigeria, Mixed [116]	2009–10	JNC 7	38.9	24.8	25.9	23.6
Nigeria, Semi-urban [117]	2011–12	≥140/90 mmHg	50	32.5	-	-
Nigeria, Urban [118]	2009–10	≥140/90 mmHg	43.88	34.8	-	-
Nigeria, Mixed [119]	2011–12	≥140/90 mmHg	41.7	31.8	33.5	30.5
Nigeria, Urban [120]	2006–07	≥140/90 mmHg	50.5	27.1	28.4	22.9
Nigeria, Rural [121]	2002–05	JNC 7	42.1	20.8	21.1	20.5
Nigeria, Urban) [122]	2007–08	≥140/90 mmHg	41.6	33	28.1	36.4
Nigeria, Rural [123]	2004–05	≥140/90 mmHg	30.7	20.2	24.8	13.2
Nigeria, Semi-urban [124]	2011	JNC 7	50.5	15	18.8	12.5
Nigeria, Mixed [125]	2007–08	≥140/90 mmHg	40.8	32.8	-	-
Nigeria, Mixed [126]	2009–10	WHO/ISH 2003	38.02	42.2	46.3	37.7
Senegal, Urban [127]	1989–90	≥140/90 mmHg	31.45	22.5	23.6	21.5
Senegal, Urban [26]	2009	≥140/90 mmHg	69.5	65.4	63.9	67.1
Togo, Urban [128]	2009–10	≥140/90 mmHg	39	26.6	25.7	27.6
Togo, Urban [129]	2011	≥140/90 mmHg	40.8	36.7	34.6	38.4

JNC: Joint National Committee on prevention, detection, evaluation, and treatment of high blood pressure, WHO/ISH: World Health Organization/International Society of Hypertension.

73% of studies were completed within a one year, with over 50% carried out in urban settings. The overall sample size from all retained studies was 197734, with a mean and median of 1958 and 1200 respectively. From all studies, the weighted mean systolic and diastolic blood pressures were 129.6 mm Hg and 78.0 mm Hg respectively. Studies were mostly conducted on people aged ≥20 years, with an estimated overall mean age of 47.4 years, ranging from 30.7 to 76 years. For the age determination of subjects across selected studies, birth certificates were mostly employed, and in the absence of valid age-verification documents, subjects' age were determined from historical landmarks.

### Prevalence and awareness rates of hypertension in Africa

Across all study settings, an elderly South African setting recorded the highest prevalence of hypertension in 2008 (77.3%, mean age 65 years) [23]. Other settings reporting higher prevalence rates of hypertension were also in older adult population surveys in Tanzania in 2010 (69.9%, mean age 76 years), Tunisia in 2003 (69.3%, mean age 69 years), and Senegal in 2009 (65.4%, mean age 69.5 years) respectively [24]–[26]. The lowest prevalence rates of hypertension were recorded in Sudan (7.5%, mean age 35 years) and Ethiopia (9.9%, mean age 36.1 years) in 1989 and 2008 respectively [27], [28] (See **Table 4** for overall study characteristics).

The pooled crude prevalence in Northern Africa was higher than in sub-Saharan Africa (SSA), with hypertension prevalence of 33.3% in Northern Africa and 27.8% in sub-Saharan Africa. In other parts of SSA, Southern Africa recorded the highest prevalence, with a prevalence of 34.6% (males 35.4%, females 34.2%). Western Africa had a prevalence of 27.3% (males 29.6, females 28.2), Central Africa recorded 21.1% (males 21.5%, females 19.3%), and Eastern Africa had 26.8% (males 25.0, females 26.1) (see **Table 5** for details). The overall pooled crude prevalence (weighted means) of hypertension for Africa was 19.7% (males 23.0%, females 20.2%) in 1990, 27.4% (males 26.9, females 28.4) in 2000, and 30.8% (males 29.7, females 31.4). There were not huge differences in hypertension prevalence between urban and rural dwellers, with the exception of 1990, where urban dwellers recorded a prevalence of 17.2% (males 21.1, females 15.1), compared to 11.1% (males 9.4%, females 8.3%) recorded among rural dwellers (with urban and rural dwellers having prevalence of 26.1% versus 26.3% in 2000 and 29.6% versus 29.0% in 2010 respectively) (see **Table 6** for details).

**Table 5:** Regional pooled hypertension prevalence rates and mean blood pressures in Africa.

Main study characteristics			Prevalence and awareness of hypertension (%)				Weighted mean blood pressure (mm Hg)	
Region	Sample size	Mean age	Both sexes (se)	Male (se)	Female (se)	Awareness rate (se)	Mean systolic BP (se)	Mean diastolic BP (se)
North	28046	54.3	33.3 (2.6)	29.6 (2.1)	35.5 (2.6)	36.2 (9.2)	129.6 (1.6)	78.0 (0.8)
SSA	169688	46.4	27.8 (1.4)	27.8 (1.6)	27.8 (1.7)	30.6 (3.1)	125.6 (0.9)	78.9 (0.5)
Central	24206	43.7	21.1 (2.7)	21.5 (3.1)	19.3 (2.9)	25.1 (4.2)	119.2 (1.8)	75.4 (0.9)
East	53312	46.0	26.8 (2.9)	25.0 (2.8)	26.1 (3.4)	40.9 (6.9)	127.5 (2.2)	79.3 (0.8)
South	34753	47.5	34.6 (4.2)	35.4 (5.1)	34.2 (4.6)	26.4 (7.0)	123.5 (2.3)	79.4 (0.9)
West	57417	46.9	27.3 (1.5)	29.6 (1.8)	28.2 (1.9)	21.7 (2.7)	128.2 (0.9)	79.8 (0.8)

SSA: sub-Saharan Africa, se: standard error.

**Table 6:** Pooled crude prevalence and awareness of hypertension from all studies.

Main study characteristics			Prevalence and awareness of hypertension (%)				Weighted mean blood pressure (mm Hg)	
Setting	Sample size	Mean age	Both sexes (se)	Male (se)	Female (se)	Awareness rate (se)	Systolic (se)	Diastolic (se)
1990								
Mixed	21416	44.1	19.7 (2.9)	23.0 (3.3)	20.2 (3.5)	16.9 (3.9)	123.8 (2.2)	77.5 (1.1)
Urban	6925	39.4	17.2 (3.5)	21.1 (4.0)	15.1 (3.5)	-	**-**	**-**
Rural	5796	48.8	11.1 (2.1)	9.4 (3.0)	8.3 (4.4)	-	**-**	**-**
2000								
Mixed	38294	51.2	27.4 (2.1)	26.9 (2.1)	28.4 (2.5)	29.2 (4.5)	126.9 (1.8)	77.2 (0.9)
Urban	20898	45.7	26.1 (1.9)	26.8 (1.5)	23.9 (1.9)	-	**-**	**-**
Rural	11377	55.2	26.3 (2.3)	24.5 (3.2)	25.7 (3.1)	-	**-**	**-**
2010								
Mixed	126754	47.1	30.8 (1.6)	29.7 (1.9)	31.4 (2.1)	33.7 (3.9)	126.9 (1.1)	79.4 (0.5)
Urban	44114	47.4	29.6 (2.1)	28.2 (2.3)	28.4 (2.5)	-	**-**	**-**
Rural	46669	48.6	29.0 (2.3)	26.9 (2.7)	30.1 (2.9)	-	**-**	**-**

se: standard error.

Across selected studies, there is evidence suggesting the awareness of hypertension among people living with the disease has been increasing since 1990; however, the overall awareness rate still remains relatively low in many parts of Africa. From the pooled analysis, a weighted awareness rate (expressed as a percentage of cases of hypertension) of 16.9% was estimated in 1990, 29.2% in 2000 and 33.7% in 2010 (see **Tables 5** **and** **6** for details).

### Modelled estimates of hypertension prevalence and number of cases in Africa

**Figure 2:**
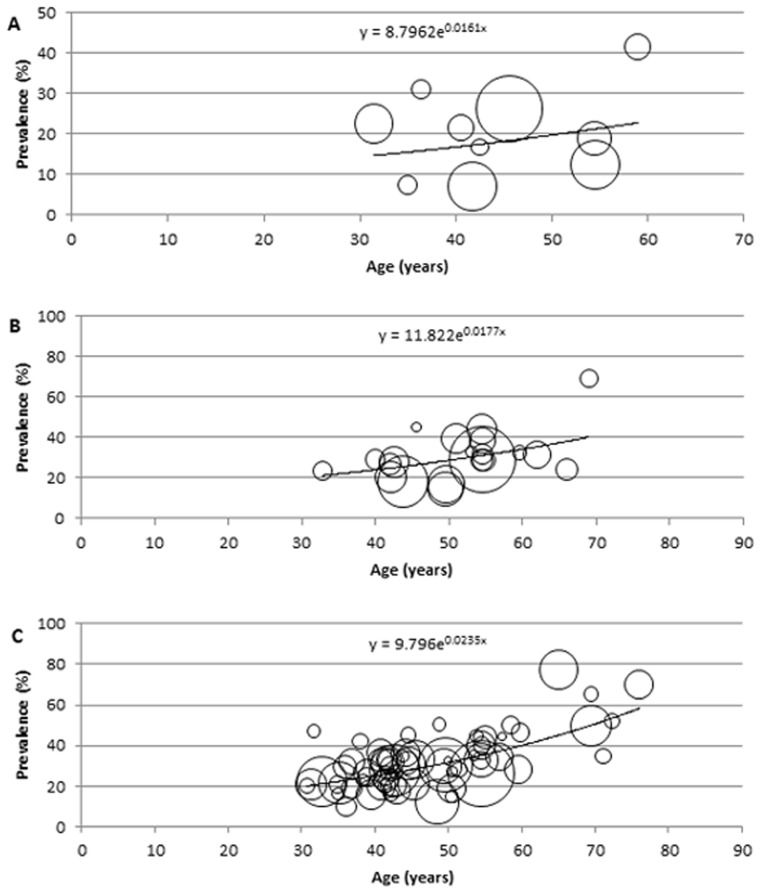
Epidemiological model showing distribution of hypertension prevalence according to age in both sexes, with size of bubble corresponding to respective sample size (A: 1990, B: 2000, C: 2010).

The modelling indicated the overall cases and prevalence of hypertension in Africa have been increasing since 1990. In adults aged ≥20 years, 54.6 million cases of hypertension were estimated in 1990 with an age-adjusted prevalence of 19.1% (13.9, 25.5), 92.3 million cases in 2000 with an age-adjusted prevalence of 24.3% (23.3, 31.6), 130.2 million cases in 2010 with an age-adjusted prevalence of 25.9% (23.5, 34.0), and a projected increase to 216.8 million cases of hypertension by 2030 with an age-adjusted prevalence of 25.3% (24.3, 39.7). The general sex distribution revealed the prevalence and number of cases of hypertension were higher among men than women. Among men, the prevalence and number of hypertension cases were both projected to increase between 2010 and 2030. About 29.8 million cases of hypertension were estimated in 1990 (21.2%, 95%CI: 16.5–29.6), 46.8 million cases in 2000 (25.1%, 95%CI: 22.9–31.0), 64.8 million cases in 2010 (26.1%, 95%CI: 23.6–33.6), and a projected increase to 112.1 million cases of hypertension by 2030 (26.4%: 24.5–41.1). However, there was a drop in prevalence among women between 2010 and 2030. 24.8 million cases of hypertension were estimated in 1990 (17.1%, 95%CI: 13.4–27.0), 45.5 million cases in 2000 (23.6%, 95%CI: 21.5–33.3), 65.4 million cases in 2010 (25.7%, 95%CI: 21.7–35.4), and a projected increase to 104.7 million cases of hypertension by 2030, with a drop in prevalence to 24.3% (95%CI: 22.4–38.9) (see **Tables 7** **–** **9** and **Figures 2** **–** **5**).

**Figure 3:**
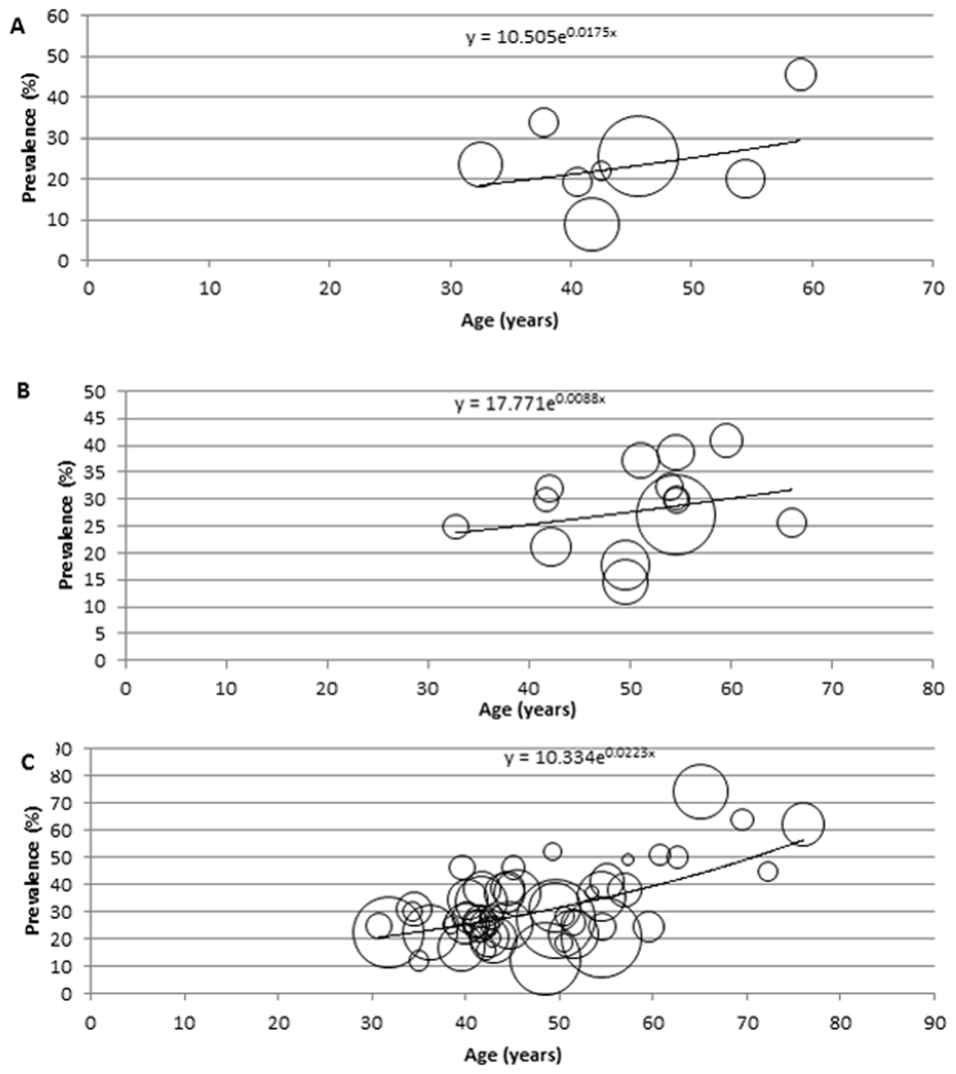
Epidemiological model showing distribution of hypertension prevalence according to age among men, with size of bubble corresponding to respective sample size (A: 1990, B: 2000, C: 2010).

**Figure 4:**
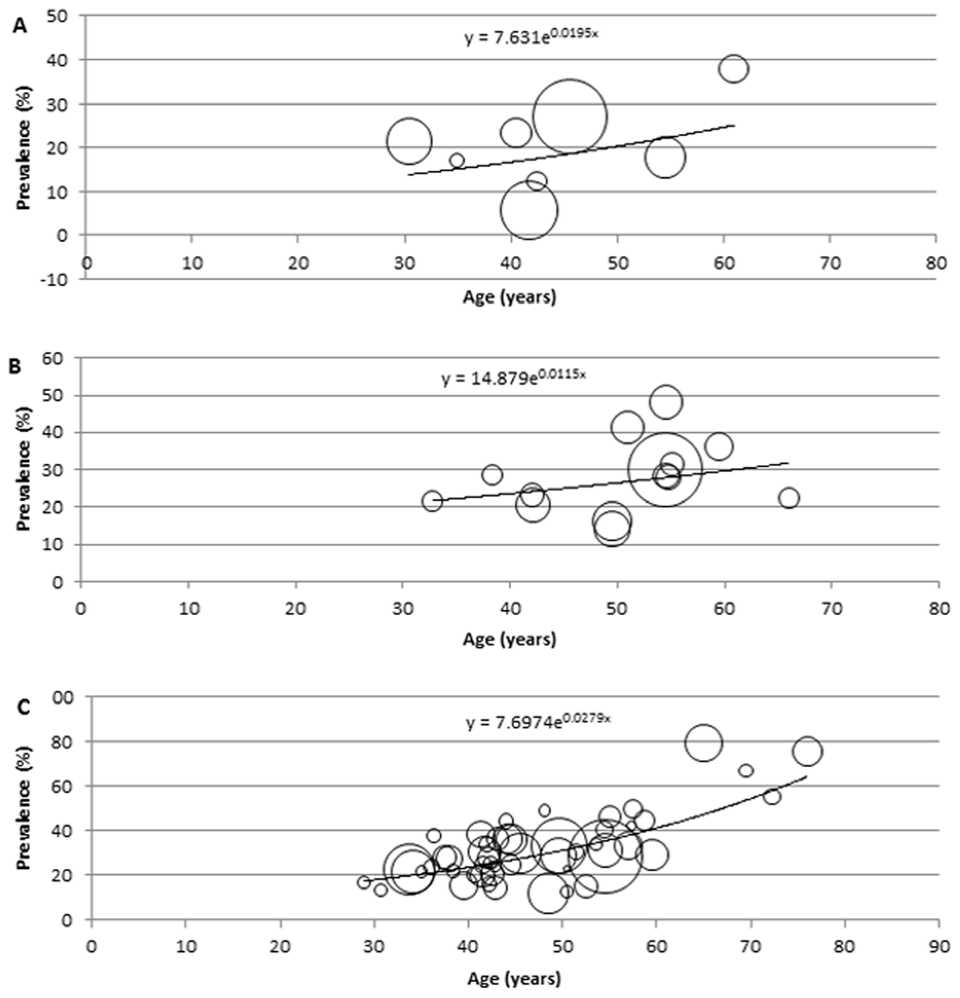
Epidemiological model showing distribution of hypertension prevalence according to age among women, with size of bubble corresponding to respective sample size (A: 1990, B: 2000, C: 2010).

**Figure 5:**
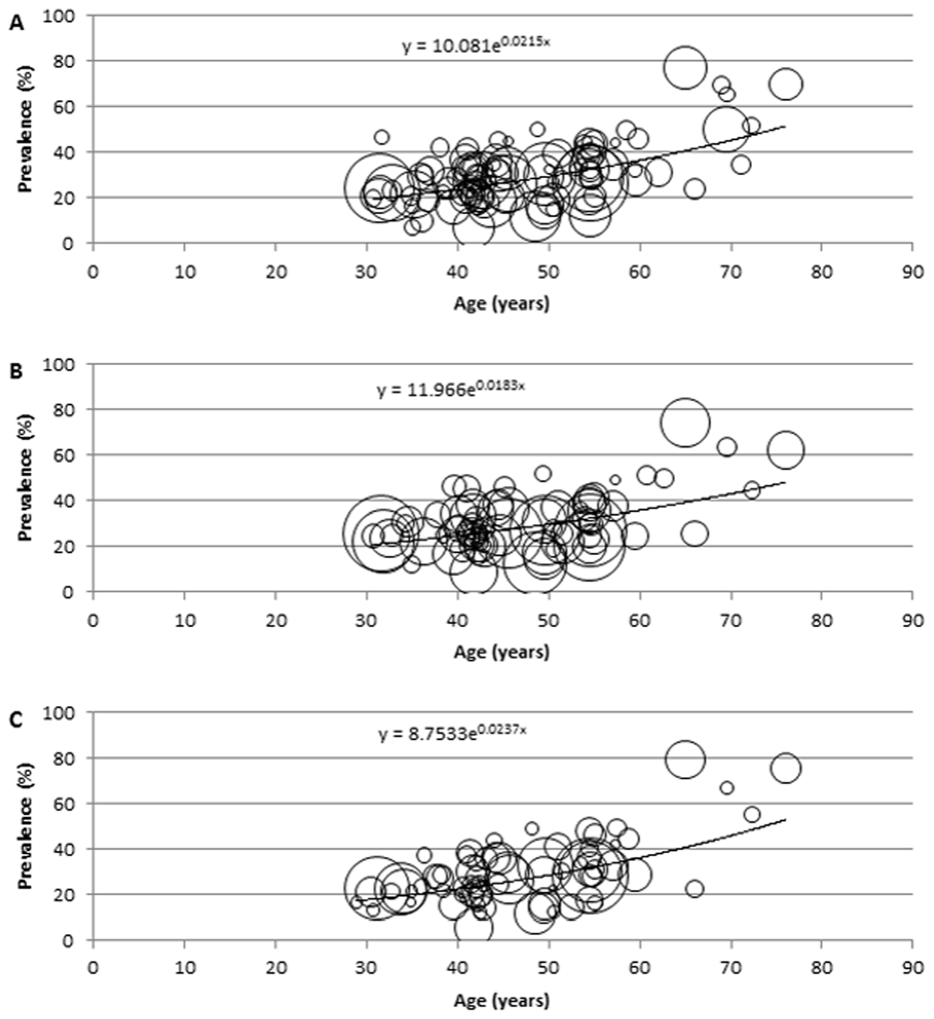
Epidemiological model showing distribution of hypertension prevalence according to age (projections for 2030), with size of bubble corresponding to respective sample size (A: both sexes, B: men, C: women).

**Table 7:** Estimated hypertension prevalence rates and cases in Africa in both sexes (estimates derived from epidemiological model and UN population demographics).

Age (years)	1990		2000		2010		2030	
	Prevalence (%) = 8.7962e^0.0161x^	Hypertension cases (000)	Prevalence (%) = 11.822e^0.0177x^	Hypertension cases (000)	Prevalence (%) = 9.796e^0.0235x^	Hypertension cases (000)	Prevalence (%) = 10.081e^0.0215x^	Hypertension cases (000)
20–24	12.5	6961.279	17.5	13134.411	16.4	15969.503	16.2	24372.870
25–29	13.6	6319.733	19.1	11761.160	18.5	15499.070	18.0	23390.362
30–34	14.7	5722.049	20.8	10540.253	20.8	14395.931	20.1	22549.551
35–39	15.9	5125.030	22.8	9662.284	23.4	12993.072	22.3	21855.722
40–44	17.3	5293.178	24.9	8844.815	26.3	11868.440	24.9	21339.520
45–49	18.7	4770.732	27.2	7956.942	29.6	11178.983	27.7	20226.663
50–54	20.3	4416.345	29.7	7076.878	33.2	10531.041	30.8	18005.650
55–59	22.0	4015.862	32.4	6135.655	37.4	9602.545	34.3	15692.831
60–64	23.9	3546.226	35.4	5372.853	42.1	8458.233	38.2	13671.611
65–69	25.9	2935.649	38.7	4436.133	47.3	6989.765	42.6	11683.620
70–74	28.0	2333.696	42.3	3326.293	53.2	5546.874	47.4	9193.002
75–80	30.4	1713.440	46.2	2078.889	59.8	3809.200	52.9	7714.551
80+	35.7	1462.513	55.1	1997.851	75.7	3327.737	65.4	7132.029
Total 20+ (95% CI)	19.1 (13.9–25.5)	54615.730	24.3 (23.3–31.6)	92324.390	25.9 (23.5–34.0)	130170.401	25.3 (24.2–39.7)	216828.010

x = mid-point of UN population 5-year age group.

**Table 8:** Estimated hypertension prevalence rates and cases in Africa among men (estimates derived from epidemiological model and UN population demographics).

Age (years)	1990		2000		2010		2030	
	Prevalence (%) = 10.505e^0.0175x^	Hypertension cases (000)	Prevalence (%) = 17.771e^0.0088x^	Hypertension cases (000)	Prevalence (%) = 10.334e^0.0223x^	Hypertension cases (000)	Prevalence (%) = 11.966e^0.0183x^	Hypertension cases (000)
20–24	15.4	4287.483	21.6	8141.867	16.9	8241.670	17.9	13606.260
25–29	16.9	3901.526	22.5	6946.029	18.9	7934.829	19.6	12816.871
30–34	18.4	3542.458	23.6	5932.465	21.1	7345.081	21.5	16039.680
35–39	20.1	3187.968	24.6	5185.153	23.6	6596.039	23.6	11574.670
40–44	21.9	2865.689	25.7	4513.438	26.4	5943.763	25.8	11090.801
45–49	23.9	2535.072	26.8	3859.029	29.5	5490.843	28.3	10275.230
50–54	26.1	2303.794	28.1	3261.431	32.9	5063.796	30.9	8962.983
55–59	28.5	2061.997	29.3	2667.812	36.8	4529.248	34.0	7618.278
60–64	31.1	1755.050	30.7	2197.312	41.2	3918.656	37.2	6406.076
65–69	33.9	1365.244	32.0	1718.733	46.0	3154.577	40.8	5243.806
70–74	37.0	978.408	33.5	1201.287	51.5	2424.530	44.7	3917.635
75–80	40.4	589.023	34.9	695.730	57.5	2238.964	48.9	2538.670
80+	48.2	411.868	38.2	464.571	71.9	1917.733	58.8	2022.895
Total 20+ (95% CI)	21.2 (16.5–29.6)	29785.580	25.1 (22.9–31.0)	46784.860	26.1 (23.6–33.6)	64799.730	26.4 (24.5–41.1)	112114.210

x = mid-point of UN population 5-year age group.

**Table 9:** Estimated hypertension prevalence rates and cases in Africa among women (estimates derived from epidemiological model and UN population demographics).

Age (years)	1990		2000		2010		2030	
	Prevalence (%) = 7.631e^0.0195x^	Hypertension cases (000)	Prevalence (%) = 14.879e^0.0115x^	Hypertension cases (000)	Prevalence (%) = 7.6974e^0.0279x^	Hypertension cases (000)	Prevalence (%) = 8.7533e^0.0237x^	Hypertension cases (000)
20–24	11.7	3253.603	19.2	7188.909	14.2	6879.980	14.7	11003.651
25–29	12.9	3018.326	20.3	6265.350	16.3	6839.885	16.6	10705.340
30–34	14.2	2791.264	21.5	5463.243	18.8	6477.360	18.7	10444.901
35–39	15.7	2548.505	22.8	4870.534	21.6	5970.342	21.0	10247.020
40–44	17.3	2320.801	24.1	4347.172	24.8	5617.575	23.7	10144.220
45–49	19.1	2108.716	25.5	3814.806	28.7	5480.796	26.7	9787.847
50–54	21.1	1978.743	27.1	3309.921	32.8	5355.558	30.0	8847.015
55–59	23.2	1800.967	28.7	2818.021	37.8	5053.976	33.8	7864.990
60–64	25.6	1593.129	30.4	2429.126	43.4	4600.255	38.0	7056.223
65–69	28.2	1289.237	32.2	1960.988	49.9	3955.928	42.8	6247.986
70–74	31.1	976.8551	34.1	1457.391	57.4	3280.345	48.2	5124.622
75–80	34.3	630.250	36.1	906.116	65.9	2970.846	54.3	3631.026
80+	41.6	519.756	40.5	707.961	87.2	2887.797	68.8	3609.290
Total 20+ years (95% CI)	17.1 (13.4–27.0)	24830.150	23.6 (21.5–33.3)	45539.541	25.7 (21.7–35.4)	65370.642	24.3 (22.4–38.9)	104714.131

x = mid-point of UN population 5-year age group.

## Discussion

This review provides an improved continent-wide estimate of the prevalence and awareness rates of hypertension in Africa using epidemiological modelling adjusted for age and sample size of the population. Having included studies conducted across various parts of Africa, the estimates may provide a close representation of the prevalence and the number of cases of hypertension in the continent.

From all studies, we estimated weighted mean systolic and diastolic blood pressures of 129.6 mm Hg and 78.0 mm Hg, respectively, with an overall mean age of 47.4 years. Our estimate is comparable with the estimates reported by Danaei and colleagues on the global trends of systolic blood pressure, with an overall mean SBP in SSA ranging 129.2–132.7 mm Hg and 132.6–134.8 mm Hg among men and women, respectively, between 1981 and 2008 [29]. This study further supports our finding of a high prevalence of hypertension in Africa, with the highest value of SBP globally estimated in SSA (along with central and eastern Europe) [29].

Across selected studies, higher prevalence rates of hypertension were reported with increasing age of subjects. This is underpinned by previous research findings where increasing age is associated with significant increase in the prevalence of hypertension, especially in people aged ≥60 years [12], [21]. For example, a higher prevalence of hypertension was reported in Northern Africa with a pooled prevalence of 33.3% compared to a prevalence of 27.8% in SSA. This could be partly explained by age difference, with a mean age of 54.3 years in Northern Africa, compared to 46.4 years in SSA. In the 2009 hospital-based Epidemiological Trial of Hypertension in North Africa (ETHNA), a high prevalence (45.4%, mean age 49.2 years) was also reported for Northern Africa [30]; this further supports findings of this study.

A higher prevalence of hypertension was noted among urban dwellers from the pooled estimates mainly in 1990 (urban 17.2%, rural 11.1%), while the prevalence was virtually the same in years 2000 and 2010. The narrowing of prevalence gaps between urban and rural dwellers in years 2000 and 2010 could be due to a possible reverse rural-urban migration, which has been reported when urban dwellers fail to cope with the economic challenges and vulnerabilities associated with urban life, and may prefer to return to natural resource-rich rural settlements [31]. In addition, there are reports that even in rural settings, the apparent remote and traditional styles do not seem to protect them again, as more of these rural settings are gradually becoming semi-urbanized [3]. Opie and colleagues also argued that many site-specific hypertension prevalence estimates in Africa may not truly reflect the burden in these settings, as there are still doubts on the proportion of Africans that truly reside in rural settings [32].

Meanwhile, an increasing, yet low, awareness rate of hypertension in Africa was reported, with pooled weighted awareness rate of 16.9% in 1990, 29.2% in 2000 and 33.7% in 2010. This estimate, to the best knowledge of our knowledge, remains the first weighted continent-wide awareness rate of hypertension reported in Africa, and thus forms an important finding of this study. The low awareness rate reported may still reflect a poor response to management of hypertension in the continent [33]. Kayima et al. corroborates this, reporting a very low awareness rate of hypertension ranging between 8% and 10% in Africa in the early 2000s [8].

In this review, the general sex distribution showed that the prevalence and cases of hypertension were higher among men than women. This is also in line with many reports in Africa [3], [8].This may be because the overall mean age from all selected studies was 47.4 years, which is just about a reported mean menopause age of 49.4 years among African women [34], and there is established evidence of a steeper blood pressure rise in men than women before the age of menopause [35]. We further estimated that there was a drop in hypertension prevalence among women between 2010 and 2030; Danaei and colleagues reported similar findings between 1981 and 2008, where, in contrast to a predominant rise in mean SBP among men, the mean SBP among women increased only in two countries globally between 1981 and 2008 [29]. Our estimate may therefore just be reflective of a continuation in this trend. Still, from the modelling, over 54.6 million cases of hypertension were estimated in 1990 (19.1%), 92.3 million cases in 2000 (24.3%), 130.2 million cases in 2010 (25.9%), and a projected increase to 216.8 million cases of hypertension by 2030 (25.3%). These estimates are higher than the 20 million reported by WHO African regional office (AFRO) in 2005 [36]. The WHO AFRO estimate was based on ≥160/95 mm Hg and this is probably the reason for the low hypertension cases reported. However, reports show that this figure has often been quoted in many official documents as the number of hypertension cases in Africa [4]. Meanwhile, Twagirumukiza et al. estimated about 75 million cases (16.2%) of hypertension among people aged ≥15 years in SSA in 2008, and projected to increase to 125.5 million cases (17.4%) in 2025 (**Table 10**) [15]. These figures are relatively low compared to the current estimates. It is understandable that these estimates were for SSA and that the mean age was low (40 years), they may yet not reflect the true burden of hypertension in the continent, as their review and analysis mainly included studies from 11 countries in Africa. In addition, another reviewer also noted these concerns, and agreed the estimates were very low compared to recent prevalence rates reported in Africa, and may be erroneously interpreted that the burden of hypertension in the continent is low [37]. However, Kearney et al. reported higher hypertension estimates, with about 79.8 million hypertension cases (27.6%) estimated among people aged ≥20 years in 2000 and projected to reach about 150.7 million cases (27.7%) in 2025 [6]. These prevalence rates are comparable with the current estimates, but the difference in the number of hypertension cases may be due to the fact that demographic changes as reported by the United Nations population projections were considered. Kearney et al. also reported a minimal change in hypertension prevalence rates between 2000 and 2025 (27.6% versus 27.7%) [6], which is also comparable with the current estimates for 2000 and 2030 (24.3% versus 25.3%) (**Table 10**). This may be due to a potentially better public health response to the overall management of hypertension in many African countries.

**Table 10:** Comparable estimates of hypertension prevalence rates from selected studies.

	Current Study*			Kearney et al.* [6]		Twagirumukiza et al.** [15]	
	2000	2010	2030	2000	2025	2008	2025
Prevalence rate (%)	24.3	25.9	25.3	27.6	27.7	16.2	17.4

*20years (all Africa),

**15+years (sub-Saharan Africa).

### Study limitations

The study aims to provide an improved continent-wide estimate of hypertension in Africa using current definitions (cut off “≥140/90 mm Hg”). However, the study has some important limitations. First, the modelling was age-dependent, we understand there are other important social and health determinants that could have resulted in varying estimates if considered, including, but not limited to the overall population characteristics, socio-economic factors and general living conditions [38]. In addition, the overall mean age was 47.4 years, which could also have resulted in a lower prevalence estimate, as research evidences show significant increase in hypertension prevalence in people aged ≥60 years [21]. Second, all studies included in our modelling were based on the blood pressure cut off “≥140/90 mm Hg”; notwithstanding, some studies have varying designs and blood pressure measuring protocols, which could have affected the quality of the current estimates. Moreover, while we ensured all studies that were graded as *high* and *moderate quality* were included in the quantitative analysis, some *low quality* studies were also included in the quantitative analysis on the basis of good study designs, which could potentially affect our overall estimates (See **Box S1**, **Table S1** and **Table S2** in **File S1**).

Third, not all studies reported age- and sex- specific estimates, including urban or rural site-specific estimates, as an epidemiology of the prevalence of hypertension in these sub-groups could have been further helpful. Furthermore, the incompleteness of data across many studies prevented us from providing estimates on the control and treatment of hypertension in Africa. We still hope that providing a continent-wide estimate of the awareness rate of hypertension may further give a general view of the public health response to the disease in the continent. However, we extracted data from 92 studies conducted in 31 African countries (having overall sample size of 197734), and with a consideration of the United Nations population demographics in the epidemiological modelling. The current estimates may therefore provide fair representation of the overall African population and better reflect the prevalence and number of cases of hypertension in the continent.

### Challenges and public health response to hypertension in Africa

Recent reports show that the World Heart Federation, supported by the Pan African Society of Cardiology (PASCAR), has been actively building capacities across Africa to address the rising burden of hypertension and other cardiovascular diseases in the continent [39]. However, collaborations and response within Africa remains poor, as hypertension still ranks low among health priorities, owing to competition for the limited resources from a co-existing high burden of infectious diseases [5]. Moreover, in countries with some levels of care for hypertension, the standards of health service delivery is poor [3]. Many cases of hypertension are detected late, treatments rarely follow standard guidelines, and the costs of medications are generally high [35]. Besides, health care seeking behaviour has also been affected, with people often preferring low-cost substandard health facilities [40]. This is particularly a problem in rural settings where the prevalence of hypertension has been reported to be on a gradual increase [8]. Further reports show that even with a relatively lower prevalence of hypertension among rural dwellers, the detection and overall management are poor in comparison to urban dwellers [3]. Additionally, many African countries are yet to implement population-wide control measures to address risk factors for hypertension [41], even with confirmed reports of high salts and fats consumption in the region and evidence showing cost-effectiveness of interventions targeting this [42], [43]. The WHO now recommends country-specific initiatives and legislation concerning food labelling and products formulation, including sodium and saturated fats content in processed foods [2], [39].

## Conclusions

This study suggests a high prevalence of hypertension in Africa, and the awareness of the disease, though increasing, still remains low. Hypertension deserves to be on the health priority lists of African nations, and problems with funding may possibly be reduced by partnering with leading international bodies for cardiovascular diseases. Essentially, policy makers and stakeholders in the health sector need to institute nationwide population-based strategies towards creating awareness on hypertension and educating people on the main risk factors such as smoking, harmful use of alcohol, sedentary lifestyles and unhealthy diets. It is hoped that the findings of this review may prompt appropriate policy response at country level towards improved detection, control and overall management of hypertension in Africa.

## Supporting Information

Checklist S1 PRISMA Checklist.
(DOC)

File S1 Box S1, Brief details of quality criteria of retained studies on hypertension in Africa. (This is a description of how studies were graded and assessed). Table S1, Quality assessment and grading of retained hypertension studies in Africa. (This shows the grading of each study). Table S2. Overall study characteristics with site identification numbers. (This shows all retained study sites with identification numbers used for grading)
(DOCX)
