# Supplementary material for: Estimating the Prevalence and Awareness Rates of Hypertension in Africa: A Systematic Analysis
Source: PLoS One. 2014 Aug 4;9(8):e104300. doi: 10.1371/journal.pone.0104300 (PMC4121276; doi:10.1371/journal.pone.0104300)
Supplement: File S1 — Box S1, Brief details of quality criteria of retained studies on hypertension in Africa. (This is a description of how studies were graded and assessed). Table S1, Quality assessment and grading of retained hypertension studies in Africa. (This shows the grading of each study). Table S2. Overall study characteristics with site identification numbers. (This shows all retained study sites with identification numbers used for grading) (DOCX) [file pone.0104300.s002.docx]

**SUPPORTING INFORMATION**

**Box S1. Brief details of quality criteria and grading of retained studies on hypertension in Africa**

For the quality grading, we adapted the Grading of Recommendations Assessment, Development and Evaluation (GRADE) guidelines [[20](#_ENREF_20)], as follows:

*High quality*: Studies with the entire three criteria listed in the methods well presented;

*Moderate quality*: Studies any two criteria, one of which must be “study design” (i.e. “study design” plus another criterion) well presented;

*Low quality*: Studies with any two criteria, or “study design” only, well represented; and

*Very low quality*: Studies with only one (excluding “study design”) or none of the three criteria well presented.

As a basic rule, all studies that were graded as *high and moderate quality* were included in the quantitative analysis. Some *low quality* studies were also included in the quantitative analysis on the basis of well-presented study designs. However, all *very low quality* studies have been excluded from the review.

Table S1. Quality assessment and grading of retained hypertension studies in Africa

| **Site ID*** | **Study design** | **Study analysis** | **Study limitations** | **Generalizability to Africa** | **Grading** |
| --- | --- | --- | --- | --- | --- |
| 1-4, 7, 10, 11, 13-16, 18-20, 22-26, 28-35, 37-41, 44-49, 51, 52, 57, 58, 60, 62, 63, 65-68, 72-77, 79-87, 90-95, 97-101 | Well explained, excluding 46 and 68 (where training/questionnaire pre-test were not clearly stated) | Well explained, excluding 60 | Well-presented across all studies | Study population representative of a larger African population across all studies | *High* |
| 5, 6, 9, 12, 17, 21, 27, 36, 42, 43, 50, 53, 59, 64, 69, 70, 78, 88, 89, 96 | Well explained, excluding 21, 64, and 78 (where there was no clear description of population survey) | Well explained, excluding 5, 6, 17, 36, 42, 50, 53, 88 and 96 | Well-presented excluding 9, 12, 27, 43, 59, 69, 70, 89 | Study population not representative of a larger African population, excluding 21, 64 and 78 that were based on elderly population groups | *Moderate* |
| 8, 54, 56, 60, 71 | Well explained | Not well explained | Not well presented | Study population not fairly representative of a larger African population | *Low* |

*see **Table S2** for details of Site ID (identification)

**Table S2**. Overall study characteristics with site identification numbers

| *Site ID* | *Country, Setting* | *Study period* | *Diagnostic criteria* | *Mean age (years)* | *Prevalence % (all)* | *Prevalence % (men)* | *Prevalence % (women)* |
| --- | --- | --- | --- | --- | --- | --- | --- |
| CENTRAL | | | | | | | |
|  | Cameroon, Mixed [[44](#_ENREF_44)] | 1995 | ≥140/90mmHg | 49.5 | 16.9 | 17.7 | 16.3 |
|  | Cameroon, Mixed [[45](#_ENREF_45)] | 1991 | ≥140/90mmHg | 41.75 | 7.07 | 8.92 | 5.69 |
|  | Cameroon, Mixed [[46](#_ENREF_46)] | 1994 | ≥140/90mmHg | 54.5 | 18.8 | 20.2 | 17.8 |
|  | Cameroon, Mixed [[46](#_ENREF_46)] | 2003 | WHO/ISH 1999 | 54.5 | 38.34 | 40.9 | 36.5 |
|  | Cameroon, Urban) [[47](#_ENREF_47)] | 2003 | WHO/ISH 1999 | 31.35 | 24.6 | 25.6 | 23.1 |
|  | Cameroon, Urban [[47](#_ENREF_47)] | 2004 | WHO/ISH 1999 | 31.35 | 20.8 | - | - |
|  | Chad, Rural [[48](#_ENREF_48)] | 2004 | WHO/ISH 2003 | 35 | 16.4 | 12.2 | 21.8 |
|  | DR Congo, Mixed [[49](#_ENREF_49)] | 2009-10 | WHO/ISH 2003 | 54.5 | 40.2 | - | - |
|  | DR Congo, Urban [[50](#_ENREF_50)] | 1983-84 | ≥140/90mmHg | 42.5 | 16.7 | 22.1 | 12.4 |
|  | Rwanda, Rural [[51](#_ENREF_51)] | 2007 | JNC 7 | 42.2 | 16.0 | 16.0 | 16.0 |
| EAST | | | | | | | |
|  | Eritea, Mixed [[52](#_ENREF_52)] | 2004 | ≥140/90mmHg | 39.5 | 16.0 | 16.88 | 15.28 |
|  | Ethiopia, Mixed [[28](#_ENREF_28)] | 2008 | JNC 7, WHO/ISH 2003 | 36.08 | 9.9 | - | - |
|  | Ethiopia, Urban [[53](#_ENREF_53)] | 2012 | JNC 7 | 51.4 | 28.3 | 26 | 30.3 |
|  | Ethiopia, Urban [[54](#_ENREF_54)] | 2009 | ≥140/90mmHg | 50.5 | 19.1 | 22 | 14.9 |
|  | Ethiopia, Urban [[55](#_ENREF_55)] | 2006 | ≥140/90mmHg | 49.5 | 30.0 | 31.5 | 28.9 |
|  | Ethiopia, Urban [[56](#_ENREF_56)] | 2009-2010 | JNC 7 | 42.9 | 17.7 | 20.0 | 14.3 |
|  | Kenya, Rural [[57](#_ENREF_57)] | 2009-11 | WHO/ISH 2003 | 40.9 | 20.2 | - | - |
|  | Kenya, Mixed [[58](#_ENREF_58)] | 2007-08 | ≥140/90mmHg | 69.5 | 50.1 | - | - |
|  | Kenya, Urban [[59](#_ENREF_59)] | 2009-09 | ≥140/90mmHg | 48.5 | 12.3 | 12.7 | 12 |
|  | Seychelles, Mixed [[60](#_ENREF_60)] | 2004 | ≥140/90mmHg | 44.5 | 31.6 | 38.4 | 24.8 |
|  | Sudan, Urban [[27](#_ENREF_27)] | 1988-89 | ≥140/90mmHg | 35 | 7.5 | - | - |
|  | Tanzania, Urban [[61](#_ENREF_61)] | 1998-99 | WHO/ISH 1999 | 54.5 | 28.9 | 27.1 | 30.2 |
|  | Tanzania, Rural [[51](#_ENREF_51)] | 2007 | JNC 7 | 42.8 | 27 | 28 | 24 |
|  | Tanzania, Rural [[24](#_ENREF_24)] | 2009-2010 | WHO/ISH 2003 | 76 | 69.9 | 62.2 | 75.8 |
|  | Tanzania, Rural [[62](#_ENREF_62)] | 1996 | WHO/ISH 1999 | 39.95 | 29.2 | 30 | 28.6 |
|  | Tanzania, Rural | 1996 | ≥140/90mmHg | 54.5 | 31.9 | 32.2 | 31.5 |
|  | Tanzania, Urban [[57](#_ENREF_57)] | 2009-11 | WHO/ISH 2003 | 36.8 | 19 | - | - |
|  | Uganda, Rural [[63](#_ENREF_63)] | 2008-09 | ≥140/90mmHg | 32.75 | 22.3 | 22.5 | 22.6 |
|  | Uganda, Rural [[64](#_ENREF_64)] | 2011 | ≥140/90mmHg | 42.5 | 20.5 | 20.7 | 20.4 |
|  | Uganda, Mixed [[65](#_ENREF_65)] | 2012 | ≥140/90mmHg | 35.15 | 21.8 | 22.3 | 21.7 |
|  | Uganda, Rural [[66](#_ENREF_66)] | 2006 | ≥140/90mmHg | 42 | 30.4 | 25.4 | 34 |
| NORTH | | | | | | | |
|  | Algeria, Rural) [[67](#_ENREF_67)] | 2010 | WHO/ISH 2003 | 58.5 | 50.2 | 51.3 | 49.7 |
|  | Algeria, Urban [[68](#_ENREF_68)] | 2004-05 | ≥140/90mmHg | 54.5 | 32.7 | 24.5 | 40.6 |
|  | Algeria, Peri-urban [[69](#_ENREF_69)] | 2006-07 | ≥140/90mmHg | 55 | 44 | 41.2 | 46.7 |
|  | Egypt, Mixed [[70](#_ENREF_70)] | 1991-93 | ≥140/90mmHg | 45.6 | 26.3 | 25.7 | 26.9 |
|  | Egypt, Rural [[71](#_ENREF_71)] | 1999-00 | ≥140/90mmHg | 42.5 | 27.9 | - | - |
|  | Morocco, Mixed [[72](#_ENREF_72)] | 2000 | ≥140/90mmHg | 51 | 39.6 | 37.2 | 41.3 |
|  | Tunisia, Mixed [[73](#_ENREF_73)] | 2004-05 | JNC 7 | 44.6 | 31.07 | 25.0 | 36.1 |
|  | Tunisia, Mixed [[74](#_ENREF_74)] | 2004-05 | ≥140/90mmHg | 49.6 | 30.6 | 27.3 | 33.1 |
|  | Tunisia, Mixed [[75](#_ENREF_75)] | 2002-03 | ≥140/90mmHg | 54.5 | 44.3 | 38.7 | 48.2 |
|  | Tunisia, Urban [[76](#_ENREF_76)] | 1995 | ≥140/90mmHg | 54.5 | 28.9 | 30 | 28.4 |
|  | Tunisia, Rural [[77](#_ENREF_77)] | 2008-09 | WHO/ISH 2003 | 72.3 | 52 | 45 | 55.5- |
|  | Tunisia, Mixed [[25](#_ENREF_25)] | 2002-03 | ≥140/90mmHg | 69 | 69.3 | - | - |
| SOUTH | | | | | | | |
|  | Angola, Urban [[78](#_ENREF_78)] | 2009-10 | JNC 7 | 44.5 | 45.2 | 46.3 | 44.2 |
|  | Angola, Mixed [[79](#_ENREF_79)] | 2011 | ≥140/90mmHg | 41.5 | 23 | 26.4 | 19.8 |
|  | Madagascar, Urban [[80](#_ENREF_80)] | 1996-97 | ≥140/90mmHg | 32.75 | 23.3 | 24.9 | 21.7 |
|  | Malawi, Rural [[51](#_ENREF_51)] | 2007 | JNC 7 | 38.4 | 23 | 24.5 | 22 |
|  | Malawi, Mixed [[81](#_ENREF_81)] | 2009 | ≥140/90mmHg | 45.5 | 33.2 | 36.9 | 29.9 |
|  | Mozambique, Mixed [[82](#_ENREF_82)] | 2005 | WHO/ISH 1999 | 54.5 | 33.1 | 35.7 | 31.2 |
|  | Namibia, Urban [[57](#_ENREF_57)] | 2009-11 | WHO/ISH 2003 | 36.9 | 32 | - | - |
|  | South Africa, Rural [[83](#_ENREF_83)] | 2004-05 | ≥140/90mmHg | 59.5 | 28.0 | 24.5 | 29.2 |
|  | South Africa, Rural [[84](#_ENREF_84)] | 2010 | ≥140/90mmHg | 54.5 | 26.2 | 20.8 | 28.5 |
|  | South Africa, Mixed [[23](#_ENREF_23)] | 2008 | ≥140/90mmHg | 65 | 77.3 | 74.4 | 79.6 |
|  | South Africa, Mixed [[85](#_ENREF_85)] | 1982 | ≥140/90mmHg | 41 | 41.6 | 45.6 | 37.75 |
|  | South Africa, Mixed [[86](#_ENREF_86)] | 1990 | ≥140/90mmHg | 40.5 | 21.5 | 19.2 | 23.4 |
|  | South Africa, Peri-urban [[87](#_ENREF_87)] | 1996 | ≥140/90mmHg | 42 | 27.1 | 31.9 | 23.4 |
|  | South Africa, Rural [[88](#_ENREF_88)] | 2002 | JNC 7 | 59.5 | 32.6 | - | - |
|  | Zambia, Urban [[89](#_ENREF_89)] | 2009-10 | WHO/ISH 2003 | 57 | 34.8 | 38 | 33.3 |
| WEST | | | | | | | |
|  | Benin, Mixed [[90](#_ENREF_90)] | 2008 | ≥140/90mmHg | 42.7 | 27.9 | - | - |
|  | Burkina Faso, Urban [[91](#_ENREF_91)] | 2004 | ≥140/90mmHg | 54.5 | 40.2 | - | - |
|  | Gambia, Mixed [[92](#_ENREF_92)] | 1998-99 | ≥140/90mmHg | 43.7 | 18.4 | - | - |
|  | Ghana, Rural [[93](#_ENREF_93)] | 2004-05 | ≥140/90mmHg | 42.4 | 25.4 | 24.1 | 25.9 |
|  | Ghana, Mixed [[94](#_ENREF_94)] | 2004 | ≥140/90mmHg | 35.9 | 29.4 | 31.04 | 28.07 |
|  | Ghana, Rural [[95](#_ENREF_95)] | 2003 | ≥140/90mmHg | 53 | 32.8 | - | - |
|  | Ghana, Mixed [[96](#_ENREF_96)] | 2001 | ≥140/90mmHg | 54.7 | 28.7 | 29.9 | 28 |
|  | Ghana, Rural [[97](#_ENREF_97)] | 2006-07 | ≥140/90mmHg | 53.5 | 35 | 37.2 | 34.1 |
|  | Ghana, Rural [[98](#_ENREF_98)] | 2002-10 | JNC 7. WHO/ISH 2003 | 66 | 24.1 | 25.7 | 22.5 |
|  | Ghana, Rural [[99](#_ENREF_99)] | 2012 | ≥140/90mmHg | 53.84 | 44.7 | - | - |
|  | Guinea, Mixed [[100](#_ENREF_100)] | 2003 | ≥140/90mmHg | 62 | 31.4 | - | - |
|  | Guinea, Rural [[101](#_ENREF_101)] | 2001 | ≥140/90mmHg | 45.5 | 45.2 | - | - |
|  | Liberia, Rural [[102](#_ENREF_102)] | 1991-92 | ≥140/90mmHg | 54.5 | 12.5 | - | - |
|  | Nigeria, Mixed [[103](#_ENREF_103)] | 2010-11 | JNC 6 | 71.1 | 34.7 | - | - |
|  | Nigeria, Semi-urban [[104](#_ENREF_104)] | 2007-08 | JNC 7 | 44.2 | 36.57 | 36.79 | 36.39 |
|  | Nigeria, Semi-urban [[105](#_ENREF_105)] | 2011-12 | JNC 7, WHO/ISH 2003 | 41.5 | 25.2 | 24.7 | 24.7 |
|  | Nigeria, Rural [[106](#_ENREF_106)] | 2010-11 | ≥140/90mmHg | 57.3 | 44.5 | 49.3 | 42.3 |
|  | Nigeria, Rural [[107](#_ENREF_107)] | 2012-13 | JNC 7 | 41.3 | 20.2 | 20.5 | 20.1 |
|  | Nigeria, Urban [[108](#_ENREF_108)] | 2006-10 | JNC 7 | 41.9 | 33 | 38.3 | 27.8 |
|  | Nigeria, Mixed [[109](#_ENREF_109)] | 2008 | JNC 7 | 48.7 | 50.5 | 52 | 49.3 |
|  | Nigeria, Rural [[110](#_ENREF_110)] | 2011 | JNC 7 | 49.7 | 13.2 | 15 | 11.9 |
|  | Nigeria, Urban [[111](#_ENREF_111)] | 1987-88 | ≥140/90mmHg | 36.35 | 31.1 | 34 | 17 |
|  | Nigeria, Mixed [[44](#_ENREF_44)] | 1995 | ≥140/90mmHg | 49.5 | 14.5 | 14.7 | 14.3 |
|  | Nigeria, Rural [[112](#_ENREF_112)] | 2005-06 | WHO/ISH 2003 | 59.8 | 46.4 | 50.2 | 44.8 |
|  | Nigeria, Semi-urban [[113](#_ENREF_113)] | 2012 | ≥140/90mmHg | 31.7 | 47 | 30.1 | 16.8 |
|  | Nigeria, Mixed [[114](#_ENREF_114)] | 2009 | ≥140/90mmHg | 34.9 | 21.1 | - | - |
|  | Nigeria, Semi-urban [[115](#_ENREF_115)] | 2002-03 | JNC 6, WHO/ISH 1999 | 55 | 21 | 23.3 | 16.4 |
|  | Nigeria, Rural [[57](#_ENREF_57)] | 2009-11 | WHO/ISH 2003 | 45.3 | 21 | - | - |
|  | Nigeria, Mixed [[116](#_ENREF_116)] | 2009-10 | JNC 7 | 38.9 | 24.8 | 25.9 | 23.6 |
|  | Nigeria, Semi-urban [[117](#_ENREF_117)] | 2011-12 | ≥140/90mmHg | 50 | 32.5 | - | - |
|  | Nigeria, Urban [[118](#_ENREF_118)] | 2009-10 | ≥140/90mmHg | 43.88 | 34.8 | - | - |
|  | Nigeria, Mixed [[119](#_ENREF_119)] | 2011-12 | ≥140/90mmHg | 41.7 | 31.8 | 33.5 | 30.5 |
|  | Nigeria, Urban [[120](#_ENREF_120)] | 2006-07 | ≥140/90mmHg | 50.5 | 27.1 | 28.4 | 22.9 |
|  | Nigeria, Rural [[121](#_ENREF_121)] | 2002-05 | JNC 7 | 42.1 | 20.8 | 21.1 | 20.5 |
|  | Nigeria, Urban) [[122](#_ENREF_122)] | 2007-08 | ≥140/90mmHg | 41.6 | 33 | 28.1 | 36.4 |
|  | Nigeria, Rural [[123](#_ENREF_123)] | 2004-05 | ≥140/90mmHg | 30.7 | 20.2 | 24.8 | 13.2 |
|  | Nigeria, Semi-urban [[124](#_ENREF_124)] | 2011 | JNC 7 | 50.5 | 15 | 18.8 | 12.5 |
|  | Nigeria, Mixed [[125](#_ENREF_125)] | 2007-08 | ≥140/90mmHg | 40.8 | 32.8 | - | - |
|  | Nigeria, Mixed [[126](#_ENREF_126)] | 2009-10 | WHO/ISH 2003 | 38.02 | 42.2 | 46.3 | 37.7 |
|  | Senegal, Urban [[127](#_ENREF_127)] | 1989-90 | ≥140/90mmHg | 31.45 | 22.5 | 23.6 | 21.5 |
|  | Senegal, Urban [[26](#_ENREF_26)] | 2009 | ≥140/90mmHg | 69.5 | 65.4 | 63.9 | 67.1 |
|  | Togo, Urban [[128](#_ENREF_128)] | 2009-10 | ≥140/90mmHg | 39 | 26.6 | 25.7 | 27.6 |
|  | Togo, Urban [[129](#_ENREF_129)] | 2011 | ≥140/90mmHg | 40.8 | 36.7 | 34.6 | 38.4 |
